# Selective Advantage of NACT in Advanced Ovarian Cancer: A Retrospective Single-Centre Analysis

**DOI:** 10.3390/medicina61081493

**Published:** 2025-08-20

**Authors:** Adrienne Szilvia Berczi, Olivér Lampé, Zoárd Tibor Krasznai, Mónika Orosz, Lili Fábián, Rudolf Lampé

**Affiliations:** Department of Obstetrics and Gynecology, Faculty of Medicine, University of Debrecen, Nagyerdei krt. 98, 4032 Debrecen, Hungary; berczi.adrienn@med.unideb.hu (A.S.B.); lampe.oliver@med.unideb.hu (O.L.); krasznai.zoard@med.unideb.hu (Z.T.K.); orosz.monika@med.unideb.hu (M.O.); lilifabian99@gmail.com (L.F.)

**Keywords:** high-grade serous epithelial ovarian cancer, neoadjuvant chemotherapy, interval debulking surgery, primary debulking surgery, surgical complexity, complete cytoreduction, surgical time

## Abstract

*Background and Objectives:* Advanced-stage epithelial ovarian cancer (EOC) is associated with poor prognosis, with complete macroscopic cytoreduction representing the strongest modifiable predictor of survival. Neoadjuvant chemotherapy (NACT) followed by interval debulking surgery (IDS) is an alternative to primary debulking surgery (PDS) in patients with high tumor burden. However, its impact on surgical complexity remains debated. This study aimed to compare operative characteristics and survival outcomes between NACT + IDS and PDS using standardized scoring metrics in a real-world oncologic setting. *Materials and Methods:* We retrospectively analyzed 47 patients with International Federation of Gynecology and Obstetrics (FIGO) stage IIIC-IV high-grade serous EOC treated between January 2018 and August 2022 at a single tertiary center. Twenty-five patients received platinum–taxane-based NACT followed by IDS, and twenty-two underwent upfront PDS with adjuvant chemotherapy. Surgical effort was quantified using the Surgical Complexity Score (SCS), and intra-abdominal tumor burden was assessed via the Peritoneal Cancer Index (PCI). Progression-free survival (PFS) and overall survival (OS) were estimated using Kaplan–Meier analysis. Hazard ratios (HRs) with 95% confidence intervals were derived from Cox proportional hazards models. *Results:* Complete cytoreduction (R0) was achieved in 76% of the NACT + IDS group and 68% of the PDS group. Mean surgical complexity and operative time were significantly lower following NACT (SCS 5.0 vs. 6.2, *p* = 0.04; 140 vs. 197 min, *p* = 0.001), without significant differences in blood loss, complication rates, or length of hospital stay. Median PFS was 25 months in the NACT + IDS group versus 21 months in the PDS group, and the difference was not statistically significant. Among patients with R0 resection, survival outcomes were comparable between treatment arms. *Conclusions:* NACT + IDS was associated with shorter and less complex surgeries in selected patients, but survival outcomes appeared similar when R0 was achieved. Data suggest that selective use of NACT in patients with extensive disease burden or limited general health status may be suitable, while confirming that complete cytoreduction remains the most critical prognostic factor, although these survival comparisons are exploratory given the retrospective design and limited sample size.

## 1. Introduction

High-grade serous epithelial ovarian cancer (EOC) is the most lethal gynecological malignancy, causing more than 200,000 deaths annually worldwide, according to GLOBOCAN 2020 estimates. Globally, it ranks eighth among causes of cancer-related mortality in women [1,2]. Its poor prognosis is mainly due to the fact that around 70% of cases are diagnosed at an advanced stage, often with extensive peritoneal dissemination [2,3]. The GLOBOCAN 2020 report projects that, by 2040, the global incidence of ovarian cancer will rise by nearly 40% and mortality by more than 50% if current trends persist [1]. These alarming trends highlight the urgent need to refine treatment strategies, particularly regarding the timing and approach of surgery.

The standard treatment for advanced-stage EOC is primary debulking surgery (PDS) followed by platinum-based adjuvant chemotherapy [4,5]. Among prognostic indicators, the extent of residual disease is the most consistent and modifiable predictor of survival. Achieving complete cytoreduction (R0) improves survival but often requires extensive surgery, increasing perioperative risks [6]. Incomplete cytoreduction (R1), where some tumor remains after the surgery, is associated with significantly worse prognosis [7,8].

For patients with high tumor burden, impaired performance status, or disease involving surgically challenging anatomical regions, neoadjuvant chemotherapy (NACT) followed by interval debulking surgery (IDS) is an alternative therapeutic strategy [9]. The rationale for NACT is that it may reduce tumor volume, thereby facilitating complete resection. However, concerns persist that chemotherapy may select chemoresistant clones and induce tissue fibrosis, potentially complicating surgical dissection and the accurate assessment of residual disease [10,11].

Large randomized trials, including EORTC 55971 and CHORUS, demonstrated non-inferiority of NACT + IDS compared to PDS in terms of progression-free survival (PFS) and overall survival (OS) [12,13]. Nevertheless, both studies have been criticized for suboptimal R0 resection rates and heterogeneity in surgical practices, which limit external validity. More recent analyses suggest that, when performed in high-volume centers, NACT + IDS may achieve R0 rates comparable to PDS [14].

The effect of NACT on surgical complexity and perioperative outcomes remains controversial. Reported reductions in surgical time following NACT range from 45 to 60 min, although some studies describe prolonged procedures due to fibrosis and dense adhesions [13,14,15]. Similarly, data on surgical complexity are conflicting: some studies report lower rates of high-complexity procedures after NACT, whereas others associate PDS with increased surgical burden [14,16,17].

Direct comparisons of surgical complexity across studies are limited due to heterogeneity in scoring systems and operative practices. Among available methods, the Surgical Complexity Score (SCS) introduced by Aletti et al. is the most widely used and provides a standardized framework for comparative evaluation [18].

In order to better understand the areas of uncertainty, we designed a retrospective single-center study to compare the impact of NACT on intraoperative parameters including surgical complexity, operative time, and blood loss, as well as perioperative outcomes and survival in patients with advanced-stage serous EOC. Our hypothesis was that neoadjuvant chemotherapy, by reducing tumor load, would lead to less complex and shorter surgeries without compromising progression-free or overall survival, compared to primary debulking surgery. To our knowledge, this is the first Hungarian single-center study to apply both the Surgical Complexity Score (SCS) and Peritoneal Cancer Index (PCI) to assess the selective impact of NACT on surgical burden and survival in high-grade serous ovarian cancer patients.

## 2. Materials and Methods

This retrospective cohort study included women diagnosed with advanced-stage (FIGO IIIC-IV) high-grade serous EOC treated at the Department of Obstetrics and Gynecology, University of Debrecen, Hungary, between January 2018 and August 2022. Patients received either PDS followed by adjuvant platinum-based chemotherapy or NACT + IDS. Eligible participants were aged ≥18 years, had an Eastern Cooperative Oncology Group (ECOG) performance status of 0–2, and were newly diagnosed with epithelial ovarian cancer (FIGO IIIC-IVB) based on radiological imaging and/or histological confirmation. During the study period, 87 patients met the initial inclusion criteria, but 9 were excluded because of disease progression after NACT, 8 because of prior malignancy, 7 for unresectable extra-abdominal metastases, and 16 for incomplete documentation or were lost to follow-up, resulting in a final analytic cohort of 47 patients.

**Figure 1:**
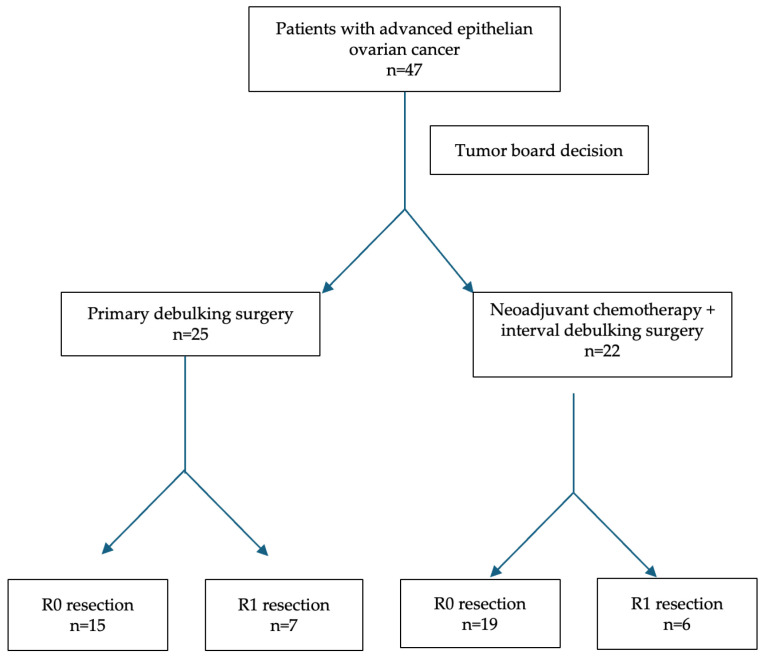
Patient flowchart. Patients with advanced-stage high-grade serous ovarian cancer (*n* = 47) were assigned to PDS or NACT + IDS based on tumor board decisions. Final surgical outcome was categorized as R0 or R1 cytoreduction.

Treatment allocation was determined by a multidisciplinary tumor board based on imaging, performance status, and diagnostic laparoscopy if needed. A comprehensive overview of the inclusion process and treatment pathway is provided in Figure 1.

All patients underwent baseline evaluation including serum Cancer antigen-125 (CA-125) measurement and contrast-enhanced thoraco-abdomino-pelvic computed tomography (CT) to determine disease extent and assess resectability.

Patients in the NACT + IDS group received three cycles of platinum–paclitaxel chemotherapy administered every 21 days. Treatment response was evaluated radiologically, and IDS was performed in cases of partial response or stable but resectable disease. Postoperatively, three additional cycles of the same regimen were administered. Patients allocated to the PDS group received six cycles of platinum-based adjuvant chemotherapy after surgery.

All surgeries were performed by midline laparotomy. Standard procedures included total abdominal hysterectomy, bilateral salpingo-oophorectomy, and total omentectomy. Additional procedures (e.g., bowel resection, diaphragmatic stripping, splenectomy, liver resection, peritonectomy, and lymphadenectomy) were carried out based on preoperative imaging and intraoperative findings.

Surgical complexity was quantified using the SCS, categorizing operations as low (1–3), intermediate (4–7), or high (≥8) complexity [18]. Intraoperative tumor burden was assessed using the PCI, which assigns scores from 0 to 3 across 13 anatomical regions, yielding a composite score ranging from 0 to 39 [19].

Statistical analyses were conducted using SPSS version 26 (IBM Corp., Armonk, NY, USA). No formal sample size calculation was performed, as this was a retrospective observational study. The final cohort size was determined by the total number of eligible patients who underwent surgery during the study period. Continuous variables were compared using Welch’s *t*-test (assuming unequal variances), while categorical variables were evaluated using Fisher’s exact test. Normality of continuous variables was assessed using Shapiro–Wilk tests and Q-Q plots. Where assumptions were questionable, Mann–Whitney U tests were run as sensitivity analyses, yielding conclusions consistent with the primary tests. Effect sizes included Cohen’s d for continuous variables, odds ratios (ORs) with 95% Wald confidence intervals (CIs) for binary outcomes, and standardized mean differences (SMDs) for covariate balance. SMDs were used to describe baseline balance; values closer to 0 indicate better balance. The number of surgical procedures per patient was analyzed using negative binomial regression to account for overdispersion, and results were expressed as rate ratios (RRs) with 95% CIs.

PFS and OS were calculated from the start of first-line treatment in both groups, corresponding to the date of surgery for the PDS group and the date of chemotherapy initiation for the NACT + IDS group, using the Kaplan–Meier method. Between-group comparisons were conducted using the log-rank test. To mitigate baseline confounding, we performed multivariable Cox proportional hazards models for PFS and OS adjusted for International Federation of Gynecology and Obstetrics (FIGO) stage (IV vs. III), age, and log-transformed CA-125. Median survival and corresponding 95% confidence intervals were estimated using the Brookmeyer–Crowley method. HRs were derived from Cox proportional hazards models (using the Breslow method for handling ties), contingent on the proportional hazards assumption, which was assessed via log-minus-log survival plots. In subgroup analyses with low event counts, incidence rate ratios (IRRs) were calculated, and statistical significance was tested using the Wald z-statistic. All statistical tests were two-sided, and a *p*-value < 0.05 was considered statistically significant. Kaplan–Meier survival curves were generated for PFS and OS. The number of patients at risk at each time point was displayed below the plots as risk tables. Between-group differences were assessed using the log-rank test, and log-rank *p*-values were reported on the plots.

The study protocol received approval from the Institutional Review Board of the University of Debrecen.

## 3. Results

A total of 47 patients were included in the study: 25 underwent NACT + IDS and 22 underwent PDS. Baseline demographic and biochemical parameters are presented in Table 1. The mean age was slightly higher in the NACT + IDS group (61.4 ± 12.4 years) than in the PDS group (57.8 ± 13.1 years), though this difference was not statistically significant (*p* = 0.34). Body mass index (BMI) was also comparable between groups (23.6 ± 3.5 vs. 24.8 ± 5.4 kg/m^2^; *p* = 0.37).

In the multivariable Cox model adjusted for FIGO stage, age, and log-transformed CA-125, NACT was not associated with significantly worse PFS (HR = 1.22; 95% CI: 0.48–3.09; *p* = 0.678), nor was FIGO IV significantly associated with PFS (HR = 1.41; 95% CI: 0.56–3.58; *p* = 0.468). In the OS model, NACT showed a non-significant trend towards improved survival (HR = 0.46; 95% CI: 0.14–1.52; *p* = 0.202), while FIGO IV showed a borderline significant association with worse OS (HR = 2.81; 95% CI: 0.88–8.99; *p* = 0.082).

Pretreatment CA-125 concentrations were significantly higher in the NACT + IDS cohort (1329.8 ± 1025.7 U/mL) than in the upfront PDS cohort (590.4 ± 1235.5 U/mL; *p* = 0.03). It is important to note that these baseline samples were taken at different clinical time points: in the NACT arm before chemotherapy, but in the PDS arm before surgery. After three cycles of neoadjuvant chemotherapy, CA-125 in the NACT group fell to 200.2 ± 287.9 U/mL. Following the completion of chemotherapies, post-treatment CA-125 levels decreased (111.2 ± 218.3 vs. 132.4 ± 486.2 U/mL), with no statistically significant difference (*p* = 0.85).

The distribution of extra-pelvic surgical procedures is detailed in Table 2. The overall number of major resections per patient was comparable between treatment arms, with a mean of 2.76 procedures in the NACT + IDS group versus 2.86 in the PDS group ([RR] = 0.96, 95% CI: 0.69–1.36; *p* = 0.83), indicating no significant difference in total surgical burden.

Large bowel resections were performed in 18% of PDS cases compared to 8% in the NACT + IDS group, and appendectomy was notably more common in the PDS cohort (32% vs. 8%). However, pelvic and upper abdominal peritonectomy rates were similar across cohorts. Other complex procedures such as retroperitoneal nodule resection, ureterectomy, and lymphadenectomy were evenly distributed between groups, further supporting comparable surgical aggressiveness in both arms.

Perioperative outcomes are summarized in Table 3. R0 was achieved in 19/25 patients (76%) after NACT + IDS and in 15/22 (68%) after PDS, a non-significant difference (OR = 1.48, 95% CI 0.41–5.33; *p* = 0.75; SMD = +0.17, represents a small effect), and showed no evidence of a real difference in R0 rates between groups. The complementary R1 rate likewise showed no separation (24% vs. 32%; OR = 0.68, 95% CI 0.19–2.44; *p* = 0.75; SMD = −0.17).

Baseline stage, however, remained imbalanced: FIGO IV disease was more common in the NACT + IDS cohort (32% vs. 9%; OR = 4.71, 95% CI 0.88–25.22; *p* = 0.08; SMD = +0.56), with a correspondingly lower proportion of FIGO III cases (68% vs. 90%; OR = 0.21; SMD = −0.56). Both SMD values exceed the 0.25 threshold, signaling residual confounding from non-random treatment allocation.

Surgical complexity clearly favored NACT: the mean SCS was 5.0 ± 1.3 versus 6.2 ± 2.4 (*p* = 0.04; Cohen’s d ≈ −0.63; SMD = −0.62). A high SCS (≥8) was observed in 16% of the NACT + IDS group (4/25) and 63.6% of the PDS group (14/22), corresponding to an OR of 0.11 (95% CI 0.03–0.43, *p* = 0.001). Operating time was significantly shorter after NACT (140 ± 41.8 min vs. 196.6 ± 63.3 min; *p* = 0.001; Cohen’s d ≈ −1.06), a large effect size.

All other intra- and postoperative metrics were comparable: estimated blood loss (242 ± 126 mL vs. 257 ± 115 mL; *p* = 0.68; d ≈ −0.12), length of stay (6.8 ± 4.5 days vs. 7.4 ± 1.9 days; *p* = 0.55; d ≈ −0.17), and mean Clavien–Dindo score (1.8 ± 0.7 vs. 1.9 ± 0.7; *p* = 0.63; d ≈ −0.14). Grade III–IV complications were equally rare (2/25 vs. 2/22; OR = 1.15; *p* = 1.00). The Peritoneal Cancer Index trended lower after NACT (6.1 ± 3.4 vs. 7.7 ± 3.7; *p* = 0.13; d ≈ −0.45).

Welch’s *t*-test was applied to continuous variables; Fisher’s exact test was applied to categorical outcomes. SMD quantifies baseline balance, with |SMD| ≤ 0.25 considered acceptable. Cohen’s d interprets the magnitude of mean differences (≈0.2 = small, 0.5 = medium, and ≥0.8 = large). Odds ratios (ORs) > 1 favor NACT + IDS and < 1 favor PDS.

**Figure 2:**
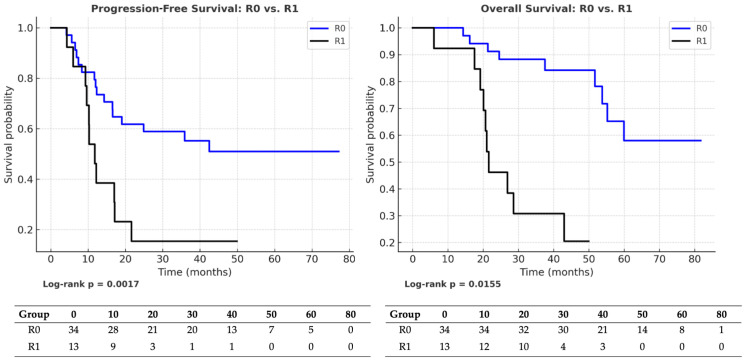
Kaplan–Meier survival analysis comparing R0 versus R1 cytoreduction in patients with advanced-stage epithelial ovarian cancer. Left panel: progression-free survival (PFS). Right panel: overall survival (OS). Risk tables indicate the number of patients at risk at each time point. Statistically significant differences were observed in both PFS (log-rank *p* = 0.0017) and OS (log-rank *p* = 0.0155) in favor of patients who achieved complete cytoreduction (R0).

The median follow-up was 37 months (IQR: 29–56). At the time of analysis, 29 progression events and 28 deaths had occurred. Among patients achieving R0 (*n* = 34), 18 experienced progression and 17 died, resulting in censoring rates of 47.1% for PFS and 50% for OS. In the R1 subgroup (*n* = 13), 11 progression events and 11 deaths were observed, yielding censoring rates of 15.4% for PFS and 15.4% for OS. R0 emerged as the most robust prognostic factor in the entire cohort. Patients who achieved R0 experienced markedly superior oncologic outcomes, with a median PFS of 36.5 months compared to 12.0 months in the R1 subgroup (HR = 0.30; 95% CI: 0.14–0.64; *p* = 0.0017). Similarly, OS was significantly longer in the R0 group (40.5 vs. 22.0 months; HR = 0.39; 95% CI: 0.18–0.84; *p* = 0.0155). Figure 2 illustrates Kaplan–Meier survival curves comparing R0 versus R1 cytoreduction. In both progression-free survival (PFS; log-rank *p* = 0.0017) and overall survival (OS; log-rank *p* = 0.0155), patients achieving R0 resection had significantly longer survival (median PFS: 36.5 vs. 12.0 months; median OS: 40.5 vs. 22.0 months). Risk tables showing the number of patients at risk at each time point are displayed below each plot.

**Figure 3:**
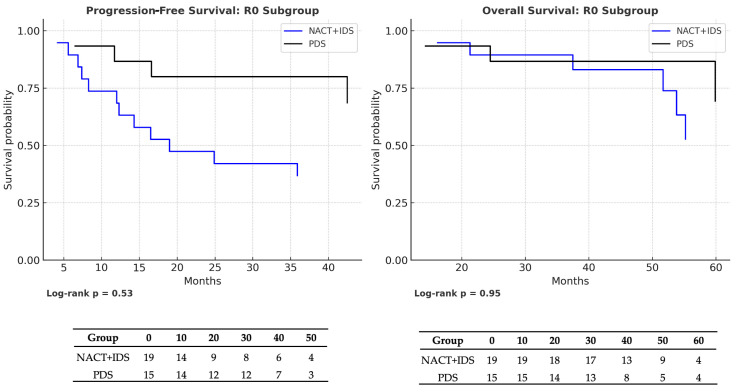
Kaplan–Meier survival curves comparing treatment modalities within the R0 subgroup: neoadjuvant chemotherapy followed by interval debulking surgery (NACT + IDS) versus primary debulking surgery (PDS). The left panel displays progression-free survival (PFS), and the right panel shows overall survival (OS). Risk tables indicating the number of patients at risk at each time point are shown below the curves. Log-rank *p*-values are reported beneath each panel. No statistically significant differences were observed between the two groups (PFS: median 39 vs. 38 months, *p* = 0.53; OS: median 54 vs. 48 months, *p* = 0.95).

When stratified by treatment modality, survival outcomes within the R0 subgroup were comparable between patients undergoing NACT + IDS and those treated with PDS. Median OS was 54 months in the NACT + IDS group and 48 months in the PDS group (HR = 0.99; 95% CI: 0.38–2.60; *p* = 0.95), while median PFS was similarly consistent (39 vs. 38 months; HR = 1.17; 95% CI: 0.45–3.01; *p* = 0.53). Kaplan–Meier survival curves with corresponding number-at-risk tables for progression-free survival (PFS) and overall survival (OS) in the R0 subgroup are shown in Figure 3. No statistically significant differences were observed between NACT + IDS and PDS in either PFS (median 39 vs. 38 months; log-rank *p* = 0.53) or OS (median 54 vs. 48 months; log-rank *p* = 0.95).

**Figure 4:**
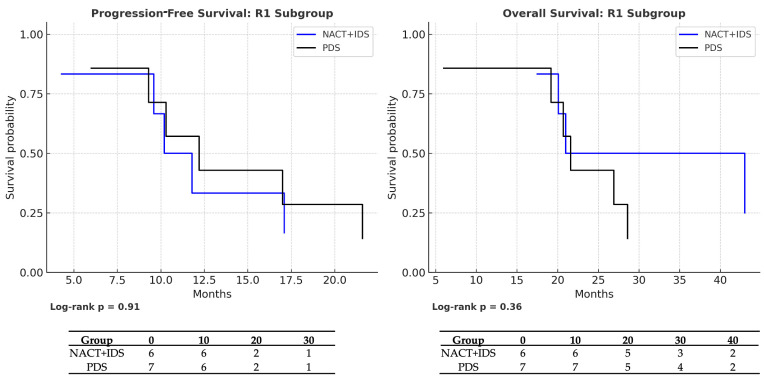
Kaplan–Meier survival curves comparing treatment modalities within the R1 subgroup: neoadjuvant chemotherapy followed by interval debulking surgery (NACT + IDS) versus primary debulking surgery (PDS). The left panel displays progression-free survival (PFS), and the right panel shows overall survival (OS). Risk tables indicating the number of patients at risk at each time point are shown below the curves. Log-rank *p*-values are reported beneath each panel. No statistically significant differences were observed between the two groups (PFS: median 10 vs. 12 months, *p* = 0.91; OS: median 21 vs. 22 months, *p* = 0.36).

Among patients with residual disease (R1), survival was similarly poor in both treatment arms, with no statistically significant differences. Median PFS was 10 months in the NACT + IDS group versus 12 months in the PDS group (HR = 0.85; 95% CI: 0.26–2.78; *p* = 0.91), and median OS was 21 vs. 22 months, respectively (HR = 0.53; 95% CI: 0.15–1.81; *p* = 0.36). Kaplan–Meier curves with corresponding number-at-risk tables are presented in Figure 4, with log-rank *p*-values reported below each panel.

The results of the univariable Cox proportional hazards analyses for progression-free survival (PFS) and overall survival (OS) in the R0 and R1 subgroups are summarized in Table 4. These analyses are consistent with the Kaplan–Meier estimates shown in Figure 2, Figure 3 and Figure 4, confirming the absence of statistically significant differences between the treatment arms in either subgroup.

## 4. Discussion

Among all prognostic factors in advanced-stage EOC, complete surgical cytoreduction remains the strongest modifiable predictor of survival. Several meta-analyses indicate a strong association between residual tumor volume and OS [20], supporting the importance of maximal-effort cytoreductive surgery, even when multivisceral resection is required. NACT + IDS was introduced as an alternative to PDS in patients where the chance of performing an R0 resection is low. Randomized trials, including EORTC 55971 and CHORUS, established the non-inferiority of NACT + IDS versus PDS in terms of OS and PFS and reported reduced perioperative morbidity in the NACT + IDS arms. However, concerns remain regarding chemotherapy-induced disease progression in a subset of patients (up to 15%), which may limit resectability or diminish the efficacy of subsequent surgery [12,13,21].

In our FIGO IIIC-IV cohort, the total number of extra-pelvic surgical procedures per patient did not differ significantly between NACT + IDS and PDS, indicating no significant difference in overall surgical burden. Pelvic and upper abdominal peritonectomy and diaphragmatic stripping rates were similar across groups, suggesting that NACT may not consistently eliminate peritoneal disease, although this observation was not statistically significant. These findings are consistent with prior reports describing the same amount of extra-pelvic surgeries in the two groups [22].

Surgical complexity was significantly lower in the NACT + IDS group, and operative duration was markedly shorter, demonstrating a large effect size. These were the only statistically significant differences observed between the treatment arms, suggesting a selective impact of NACT on surgical parameters. No significant differences were found in blood loss, length of hospital stay, complication severity, or PCI, indicating that the effect of NACT + IDS may be confined to operative efficiency rather than overall surgical morbidity. Our findings are consistent with some previous studies [15]. This concordance with large randomized controlled trials reinforces the validity of our observations; however, the retrospective nature of our study should be noted. Our operative time results suggest that neoadjuvant therapy can, in many cases, facilitate technically easier surgery. The advantage of shorter operative times lies in reduced anesthetic exposure and decreased metabolic stress associated with prolonged procedures.

Estimated blood loss, duration of hospitalization, and the incidence of Clavien–Dindo grade III-IV complications were comparable across groups. Our complication rates are different from the EORTC 55971 trial, which reported fewer complications with NACT. Instead, our findings align with more recent meta-analyses suggesting that the perioperative benefit of NACT decreases once baseline differences are considered [23].

Consistent with prior studies, complete cytoreduction was associated with significantly better outcomes in our cohort [7]. Patients achieving R0 resection had markedly improved survival compared to R1. These findings confirm the central role of R0 status in determining prognosis.

Our results show that treatment sequencing did not impact survival among patients who achieved R0 resection. Median OS was similar in the NACT + IDS and PDS groups, as was PFS. Among R1 patients, outcomes were comparable across arms, with no significant differences in OS or PFS. These observations are consistent with results from EORTC 55971 and CHORUS, suggesting that the oncologic benefit of NACT lies in enabling resectability rather than in altering tumor biology [12,13].

This study has several limitations. Its retrospective, single-center design inherently introduces selection bias and restricts generalizability. Treatment allocation was not randomized, and patients with more extensive disease (FIGO stage IV) were more likely to receive NACT + IDS, resulting in a baseline imbalance between groups. We addressed this by adjusting for FIGO stage, age, and CA-125 in multivariable survival models, which did not materially alter the conclusions. Nonetheless, this imbalance limits cross-group comparability, and survival comparisons should be regarded as exploratory. Surgical Complexity Scores were assigned based on operative reports, which may be subject to inter-observer variability. The relatively small sample size, while sufficient for descriptive and operative endpoints, limits the statistical power to detect significant differences in survival outcomes. Consequently, survival results should be interpreted as exploratory and hypothesis-generating. As a result, several potentially meaningful differences did not meet conventional thresholds for statistical significance and should be interpreted with caution. This study was not powered to detect non-inferiority in survival outcomes between the two approaches. The median follow-up duration of 37 months may also limit the detection of late recurrences or survival plateaus. A potential source of immortal time bias arises from the use of different survival time origins: surgery date for PDS and chemotherapy initiation for NACT. Although we calculated survival from the initiation of first-line treatment in both arms and not from the interval debulking date in the NACT group, the asymmetrical time origins may still introduce bias and favor the PDS group. This limitation should be taken into account when interpreting survival differences.

In summary, in this FIGO IIIC-IV cohort, NACT + IDS was associated with significantly reduced operative time and surgical complexity but did not affect overall cytoreduction rates, postoperative morbidity, or survival compared to PDS. R0 cytoreduction remained the dominant prognostic factor regardless of treatment sequence, reinforcing the principle of maximal-effort cytoreduction. The differences observed in operative parameters suggest that NACT may improve specific aspects of surgical efficiency, particularly operative time and complexity, in selected patients. These are typically individuals with a high tumor burden or limited performance status who might benefit most from tumor load reduction prior to surgery. Strengths of this study include a homogeneous surgical approach by a high-volume team, comprehensive data collection, and the use of validated scoring systems. Despite its limitations, this study contributes clinically relevant data that may assist in refining patient selection for neoadjuvant strategies and underscores the need for prospective trials to better define the indications and oncologic impact of NACT followed by IDS in advanced ovarian cancer.

## 5. Conclusions

In this FIGO IIIC–IV cohort, NACT + IDS reduced operative time and surgical complexity but did not affect cytoreduction rates, postoperative morbidity, or survival compared with PDS. Complete cytoreduction remained the strongest prognostic factor regardless of treatment sequence. NACT may offer operative efficiency benefits for selected patients with high tumor burden or limited performance status. These findings, while limited by the study’s retrospective design and sample size, support careful patient selection for neoadjuvant strategies and highlight the need for prospective trials to clarify their oncologic impact.

## Figures and Tables

**Table 1:** Baseline characteristics of the study groups.

Variable	NACT + IDS (*n* = 25)	PDS (*n* = 22)	*p*-Value
Mean age, years	61.4 ± 12.4	57.8 ± 13.1	0.34
BMI (kg/m^2^)	23.6 ± 3.5	24.8 ± 5.4	0.37
CA-125 (U/mL), pre-treatment	1329.8 ± 1025.7	590.4 ± 1235.5	**0.03**
CA-125 (U/mL), post-treatment	111.2 ± 218.3	132.4 ± 486.2	0.85

Values are presented as mean ± standard deviation. Statistically significant differences are shown in bold.

**Table 2:** Distribution of major surgical procedures performed during cytoreduction in NACT + IDS and PDS groups.

Surgical Procedure	NACT + IDS (*n* = 25), *n* (%)	PDS (*n* = 22), *n* (%)
Wedge resection (liver)	0 (0%)	1 (5%)
Stripping diaphragm/ falciform ligament	5 (20%)	5 (23%)
Splenectomy	0 (0%)	1 (5%)
Mesenteric resection	5 (20%)	3 (14%)
Large bowel resection	2 (8%)	4 (18%)
Small bowel resection	1 (4%)	2 (9%)
Ileocecal resection/ right hemicolectomy	1 (4%)	0 (0%)
Appendectomy	2 (8%)	7 (32%)
Stoma formation	1 (4%)	1 (5%)
Lesser omentum/ stomach resection	4 (16%)	1 (5%)
Cholecystectomy	1 (4%)	0 (0%)
Pelvic peritonectomy	17 (68%)	15 (62%)
Bladder peritonectomy	9 (36%)	9 (41%)
Upper abdominal peritonectomy	9 (36%)	5 (22%)
Retroperitoneal wall resection	2 (8%)	1 (5%)
Para-aortic lymph node dissection	3 (12%)	3 (14%)
Pelvic lymph node dissection	6 (24%)	5 (23%)
Ureter resection	1 (4%)	0 (0%)
Total number of procedures performed	69	63

Multiple procedures may have been performed for the same patient; therefore, the total count of procedures exceeds the number of patients. Percentages refer to the proportion of patients within each treatment group who underwent the given procedure.

**Table 3:** Surgical and perioperative characteristics in patients undergoing NACT + IDS or PDS.

Variable	NACT + IDS (*n* = 25)	PDS (*n* = 22)	*p*-Value	SMD	Effect Size
Oncologic characteristics					
R0, *n* (%)	19 (76)	15 (68)	0.75	+0.17	OR 1.48 (0.41–5.33)
R1, *n* (%)	6 (24)	7 (32)	0.75	−0.17	OR 0.68 (0.19–2.44)
FIGO stage III, *n* (%)	17 (68)	20 (90)	0.08	−0.56	OR 0.21 (0.04–1.14)
FIGO stage IV, *n* (%)	8 (32)	2 (9)	0.08	+0.56	OR 4.71 (0.88–25.22)
Surgical parameters					
Surgical Complexity Score (mean ± SD)	5.0 ± 1.3	6.2 ± 2.4	**0.04**	−0.62	Cohen’s d = −0.63
Low SCS (1–3), *n* (%)	5 (20)	1 (4.5)			
Intermediate SCS (4–7), *n* (%)	16 (64)	7 (31.8)			
High SCS (≥8), *n* (%)	4 (16)	14 (63.6)	**0.001**	–	OR 0.11 (0.03–0.43)
Peritoneal Cancer Index (mean ± SD)	6.1 ± 3.4	7.7 ± 3.7	0.13	−0.45	Cohen’s d = −0.45
Operating time, min (mean ± SD)	140 ± 41.8	196.6 ± 63.3	**0.001**	−1.06	Cohen’s d = −1.06
Estimated blood loss, mL (mean ± SD)	242 ± 126.4	256.8 ± 114.7	0.68	−0.12	Cohen’s d = −0.12
Perioperative outcomes					
Hospital stay, days (mean ± SD)	6.8 ± 4.5	7.4 ± 1.9	0.55	−0.17	Cohen’s d = −0.17
Clavien–Dindo score, mean ± SD	1.8 ± 0.7	1.9 ± 0.7	0.63	−0.14	Cohen’s d = −0.14
Clavien–Dindo I–II/III–IV, *n*/*n*	23/2	20/2	1.00	–	OR 1.15 (0.15–8.93)

SMD: standardized mean difference; OR: odds ratio (95% CI shown in parentheses); Cohen’s d: standardized effect size. Continuous variables were compared with Welch’s *t*-test and categorical variables with Fisher’s exact test (2 × 2). SMD ≤ 0.25 indicates adequate baseline balance. A high SCS (≥8) vs. all others was compared with Fisher’s exact test. Statistically significant differences are shown in bold.

**Table 4:** Summary of survival comparisons.

Comparison	Group/Outcome	HR (95% CI)	*p*-Value	Median (Months)	Events/Total (Censoring %)
R0 → R1	PFS (entire cohort)	0.30 (0.14–0.64)	**0.0017**	R0: 36.5 vs. R1: 12	R0: 18/34 (47%) vs. R1: 11/13 (15%)
	OS (entire cohort)	0.39 (0.18–0.84)	**0.0155**	R0: 40.5 vs. R1: 22	R0: 17/34 (50%) vs. R1: 11/13 (15%)
NACT → PDS (R0 subgroup)	PFS	1.17 (0.45–3.01)	0.53	NACT: 39 vs. PDS: 38	NACT: 10/19 (47.4%) vs. PDS: 8/15 (46.7%)
	OS	0.99 (0.38–2.60)	0.95	NACT: 54 vs. PDS: 48	NACT: 9/19 (52.6%) vs. PDS: 8/15 (46.7%)
NACT → PDS (R1 subgroup)	PFS	0.85 (0.26–2.78)	0.91	NACT: 10 vs. PDS: 12	NACT: 5/6 (16.7%) vs. PDS: 6/7 (14.3%)
	OS	0.53 (0.15–1.81)	0.36	NACT: 21 vs. PDS: 22	NACT: 5/6 (16.7%) vs. PDS: 6/7 (14.3%)

HR: hazard ratio (95% CI shown in parentheses); *p*-values derived from the Wald statistic of the Cox proportional hazards model (or the log-rank test where appropriate). HR < 1 favors the first-listed (reference) group; HR > 1 favors the second. Medians are Kaplan–Meier estimates. Censoring % = (1 − events/total) × 100. Statistically significant differences are shown in bold.

## Data Availability

The data presented in this study are available by contacting the corresponding author.

## Abbreviations

The following abbreviations are used in this manuscript:

EOC	Epithelial ovarian cancer
NACT	Neoadjuvant chemotherapy
IDS	Interval debulking surgery
PDS	Primary debulking surgery
FIGO	International Federation of Gynecology and Obstetrics
SCS	Surgical Complexity Score
PCI	Peritoneal Cancer Index
PFS	Progression-free Survival
OS	Overall survival
HR	Hazard ratio
R0	Complete cytoreduction
R1	Incomplete cytoreduction
ECOG	Eastern Cooperative Oncology Group
CA-125	Cancer antigen-125
CT	Computed tomography
OR	Odds ratio
CI	Confidence interval
SMD	Standardized mean differences
RR	Rate ratio
IRR	Incidence rate ratios
BMI	Body mass index
